# Perceived Stress and Burnout Among Nurses in Acute and Critical Care Settings: The Mediating Role of Self‐Efficacy

**DOI:** 10.1111/nicc.70364

**Published:** 2026-02-01

**Authors:** Shu‐Yen Lee, Chung‐Yi Li, Peng‐Ching Hsiao, Chieh‐Yi Song, Chia‐Huei Lin, Kai‐Jo Chiang, Hsueh‐Hsing Pan

**Affiliations:** ^1^ Department of Nursing Tri‐Service General Hospital Taipei Taiwan; ^2^ College of Nursing, National Defense Medical University Taipei Taiwan; ^3^ Department of Public Health, College of Medicine National Cheng Kung University Tainan Taiwan; ^4^ Department of Public Health, College of Public Health China Medical University Taichung Taiwan; ^5^ Department of Healthcare Administration, College of Medical and Health Science Asia University Taichung Taiwan

**Keywords:** burnout, mediator, nurse, perceived stress, self‐efficacy

## Abstract

**Background:**

Nurses working in acute and critical care settings, including general wards, intensive care units and emergency departments, are exposed to high job demands that increase the risk of stress and burnout. Self‐efficacy has been identified as an important psychological resource that may buffer these effects; yet limited evidence has explored its mediating role in clinical nursing practice.

**Aim:**

To examine the association relationships between perceived stress, self‐efficacy, and burnout among hospital nurses and to test the mediating role of self‐efficacy, with subgroup analysis across demographic and work‐related factors.

**Study Design:**

A cross‐sectional design was conducted through quota sampling to recruit registered nurses from a medical centre in Taiwan between July and October 2020. Data were collected using a self‐report questionnaire including demographic and work‐related characteristics, the Perceived Stress Scale, the General Self‐Efficacy Scale and the Burnout Scale. Descriptive analyses and mediation with bootstrapping were used to test the indirect effect of self‐efficacy, with stratified analyses for subgroups.

**Results:**

Of the 395 nurses invited, 380 completed the survey, yielding a response rate of 96.2%. Higher perceived stress was significantly associated with lower self‐efficacy, which in turn increased burnout. Mediation analysis confirmed that self‐efficacy played a significant indirect role in the stress–burnout pathway. The mediating effect was particularly evident among nurses aged over 40 years and those working day shifts, groups often holding leadership or supervisory responsibilities in acute and critical care environments.

**Conclusions:**

Self‐efficacy is a key psychological mechanism linking stress to burnout among nurses in acute and critical care settings. This study adds evidence that reinforcing self‐efficacy may reduce vulnerability to burnout, particularly among senior and supervisory nurses.

**Relevance to Clinical Practice:**

Interventions designed to reduce perceived stress while enhancing self‐efficacy, such as resilience training, simulation‐based education and mentorship programmes may help mitigate burnout, improve nurse retention and support well‐being in high‐intensity clinical settings.

## Impact Statements

- What is known about this topic?
  ○ Nurses in acute and critical care settings are at high risk of stress and burnout due to demanding workloads and complex patient care.
  ○ Burnout negatively affects nurse well‐being, retention, and patient outcomes.
  ○ Self‐efficacy has been identified as a protective factor, but its mediating role in the stress–burnout relationship remains unclear.
- What this paper adds?
  ○ Demonstrates that self‐efficacy significantly mediates the association between perceived stress and burnout among nurses.
  ○ Identifies nurses over 40 years and those working day shifts, as particularly vulnerable.
  ○ Highlights the need for targeted interventions that enhance self‐efficacy to reduce burnout and support workforce sustainability in acute and critical care environments.

## 1 Introduction

The global nursing shortage significantly impacts work environments, contributing to elevated turnover rates and intensifying challenges for healthcare systems. Insufficient resources and excessive demands in the workplace increase nurses' workloads, escalating levels of stress and burnout among nurses [1]. Recent studies reveal that over 40% of nurses experience burnout [2, 3], highlighting its prevalence as a critical concern worldwide [4, 5].

Burnout is a psychological syndrome characterised by emotional exhaustion, depersonalisation and diminished personal achievement. It develops when individuals face persistent, unrelenting stress that depletes energy, reduces motivation and decreases commitment to work [6, 7]. For nurses, burnout not only undermines personal well‐being but also negatively impacts hospitals and patient outcomes [8]. Factors contributing to nurse burnout include substantial workload, exposure to death and suffering, hierarchical challenges, treatment uncertainties, frequent temporary transfers due to staff shortages and care for complex patients [2, 8, 9]. The escalating demand placed on nurses amplifies their vulnerability to burnout, making it essential to better understand and address this issue, especially as workforce sustainability becomes increasingly critical [8].

Theoretical models, such as the Transactional Model of Stress and Coping [10], Bandura's Self‐Efficacy Theory [11], the Job Demands‐Resources (JD‐R) Model [12, 13] and Hobfoll's Conservation of Resources (COR) Theory [14], have been developed to explain the multifactorial causes of burnout. These models identify key factors such as perceived stress and self‐efficacy. However, little is known about the complex interactions between these factors, which could reveal both risk and protective factors for burnout and inform more effective interventions.

Perceived stress is defined as the subjective appraisal of stress levels when environmental demands exceed an individual's available resources, adversely affecting physical and psychological well‐being [15]. The extent of perceived stress varies depending on how individuals interpret and respond to external challenges [16]. Nurses working in complex and demanding environments often face elevated levels of perceived stress, increasing their susceptibility to burnout and prompting some to consider leaving the profession [17, 18]. A previous study indicates a significant positive correlation between perceived stress and burnout among nurses [2].

Self‐efficacy, defined as the ‘belief in one's capabilities to organise and execute the courses of action required to achieve specified attainments’ [19], is a critical psychological construct that empowers individuals to manage stressful demands with confidence [20]. Evidence suggests that self‐efficacy not only reduces perceived stress but also acts as a protective buffer against burnout [21, 22]. Studies indicate that self‐efficacy negatively correlates with burnout, while perceived stress has a positive association with burnout and a negative relationship with self‐efficacy [23, 24]. By exploring self‐efficacy as a mediator, this study aims to explore its role in mitigating burnout through perceived stress.

Despite extensive research, most studies have examined perceived stress, self‐efficacy, and burnout independently, leaving their complex interactions among nurses underexplored. The applicability of findings from other healthcare professionals to nursing remains uncertain, and the mechanisms underlying these associations are not well defined. Although perceived stress is consistently linked to higher burnout, the strength of this relationship may vary across individuals and work contexts, suggesting both direct and indirect pathways. Understanding these dynamics in acute and critical care settings is essential for developing effective interventions. To address these gaps, this study investigates whether self‐efficacy mediates the relationship between perceived stress and burnout among nurses in acute and critical care settings, providing evidence to guide targeted strategies that enhance resilience and workforce well‐being.

## 2 Design and Methods

### 2.1 Study Design

This study employed a cross‐sectional design, and its reporting adhered to the Strengthening the Reporting of Observational Studies in Epidemiology (STROBE) checklist [25].

### 2.2 Setting and Sample

This study was conducted at a medical centre in northern Taiwan, comprising approximately 1700 beds, 24 medical and surgical wards, six intensive care units (ICUs) and one emergency room (ER). During the study period in 2020, a total of 1800 nurses were employed at this medical centre. Quota sampling was applied based on the proportional representation of nurses working in medical and surgical wards, ICUs and ER. Participants were recruited between July and October 2020. The inclusion criteria were registered nurses employed at the medical centre who agreed to participate in the study. Nurses who were unwilling to participate were excluded from the study.

Sample size estimation was performed using G*Power 3.1.9.2 for linear bivariate regression with a one‐group design. Assuming a two‐tailed test, a statistical power of 95% and the Type I error (alpha) of 0.05 [26], the minimum required sample size was 314 participants, indicating adequate statistical power for the analyses.

### 2.3 Data Collection Tools

The questionnaires used in this study included demographics and work‐related characteristics, the Perceived Stress Scale (PSS‐14), General Self‐Efficacy Scale (GSES) and Burnout Scale (BS).

Demographic characteristics assessed included age, sex, educational level, religious beliefs, marital status and level of family support, which were rated on a scale of 1–5, with 1 indicating very low support and 5 indicating very high support from nurses' family members. Work‐related characteristics were considered, including the working ward (general ward, ICU or emergency room), length of nursing service, shift timing (fixed day, night, or rotating shifts) and participation in stress‐related education.

#### 2.3.1 Perceived Stress Scale (PSS‐14)

The PSS‐14 is a widely used psychological instrument to assess stress perception, focusing on unpredictability, uncontrollability and overload, reflecting an individual's stress over the past month. The Taiwanese translation demonstrates satisfactory validity and reliability [27]. The scale comprises 14 items evenly divided into seven positive and seven negative statements. The respondents rated each item on a scale of 0–4, where 0 denoted ‘never’ and 4 denoted ‘always’. Positive item scores were converted to calculate the total score, resulting in a possible range of 0 to 56, with higher scores indicating higher perceived stress levels. In this study, the Cronbach's alpha for the PSS‐14 was 0.787.

#### 2.3.2 General Self‐Efficacy Scale

The Chinese version of the General Self‐Efficacy Scale (GSES) was originally developed by Schwarzer, and is routinely employed to assess individuals' self‐confidence in their responses to various challenges or encounters with novel circumstances [28]. The GSES comprises 10 items, each assessed on a 4‐point Likert scale ranging from 1 (‘not at all true’) to 4 (‘exactly true’). A single score for the scale is derived by calculating the mean of all items. The total score ranges from 1 to 4, with a higher score indicating a higher level of general self‐efficacy. The Chinese version of the GSES has been adapted for nurses and has shown good reliability and validity [29]. In this study, the Cronbach's alpha for the GSES was 0.877.

#### 2.3.3 Burnout Scale

Burnout was assessed using Stamm's Burnout Scale (BS), a subscale derived from the Professional Quality of Life Scale (ProQOL, Version 5) [30]. The 10‐item scale measures emotional exhaustion, depersonalisation or cynicism, and perceived inefficacy on a 5‐point Likert scale ranging from 1 (‘never’) to 5 (‘always’), with higher scores indicating greater burnout. Burnout levels were categorised into three groups based on quartiles: low (< 25%), moderate (25%–75%) and high (> 75%). The instrument has demonstrated satisfactory internal consistency across healthcare populations, with reported reliability coefficients ranging from 0.75 to 0.85 [31]. In this study, the Cronbach's alpha for the BS was 0.799, indicating acceptable reliability for psychological research instruments with a limited number of items.

### 2.4 Study Procedure

After obtaining IRB approval, the project investigator (PI) contacted the head nurses of the Nursing Department. During these interactions, the PI provided a comprehensive explanation of the purpose and methodology of the study. Nurses who met the inclusion criteria were invited to participate and every participant should provide their consent. Data were collected using questionnaires, with each participant dedicating approximately 15 min to completing the study. Confidentiality of the data was ensured and the participants had the right to withdraw from the study at any time.

### 2.5 Data Analysis

Data were organised and stored in Microsoft Excel, and the analysis was conducted using IBM SPSS Statistics for Windows 20.0 [32]. For continuous variables, descriptive statistics such as mean and standard deviation (SD) were utilised, whereas categorical variables were described using frequency and percentage. Bivariate correlations were used to explore the relationships among perceived stress, self‐efficacy and burnout. Statistical significance was set at *p* < 0.05.

To address the issue of multiple testing inherent in the examination of bivariate correlations among the study variables, the Bonferroni correction was implemented. A series of multiple regression analyses was performed to evaluate different aspects of the relationships among the variables and to specifically assess the mediating effects of self‐efficacy on the relationship between perceived stress and burnout. These analyses examined the associations between perceived stress and self‐efficacy (*a* path), self‐efficacy and burnout (*b* path) and perceived stress and burnout (*c* path, denoted as ‘total effects’), all while accounting for relevant covariates. Moreover, we computed the direct effects of perceived stress on burnout, taking into consideration self‐efficacy and other covariates (*c'* path). To ascertain the significance of the mediation effects, as defined by the product of the regression estimates for the *a* and *b* paths, we employed a bootstrapping method with 1000 bootstrapped samples based on computational feasibility and prior methodological literature indicating that this number yields reliable estimates for moderate to large mediation effects. A mediation effect was considered significant when the 95% bias‐corrected bootstrap confidence interval (CI) did not encompass 0. Additionally, a stratified analysis was employed to investigate the proportion of the indirect effect of self‐efficacy on the association between perceived stress and burnout within the subgroups defined by each variable. As multiple subgroup comparisons (age, shift type and education level) were performed, the potential for Type I error was recognised and subsequently addressed as a study limitation.

### 2.6 Ethical and Institutional Approvals

In accordance with the Declaration of Helsinki, this study received ethical approval from the Institutional Review Board (IRB) of the medical centre (IRB No. B202005010), approved on 29 April 2020.

## 3 Results

### 3.1 Demographics and Work‐Related Characteristics

A total of 395 nurses were recruited for this study, of whom 380 completed the survey, yielding the response rate of 96.2%. The nurses' mean age was 31.8 years (range = 20–59 years). Most were under 30 years old (54.7%), female (93.9%), held a bachelor's degree (72.6%), reported no religious beliefs (51.8%) and were married (70.8%). Family support had an average score of 4.2 (SD = 0.8) on a scale ranging from 1 (very low support) to 5 (very high support), and most nurses reported having high family support (80.5%).

In terms of work‐related characteristics, most nurses worked in the ICU (53.2%), worked only night shifts (45.8%) and 53.4% did not participate in stress‐related education. The average number of years of nursing service was 8.8 years (SD = 8.5) (Table 1).

**TABLE 1:** Demographics and work‐related characteristics among nurses (*N* = 380).

Variable	Mean (SD)/*N* (%)
Demographics	
Age (year)	31.8 (8.4)
< 30 years	208 (54.7)
30–40 years	106 (27.9)
> 40 years	66 (17.4)
Gender	
Female	357 (93.9)
Male	23 (6.1)
Educational Level	
Junior college	43 (11.3)
Bachelor	276 (72.6)
Master	61 (16.1)
Religious Belief	
No	197 (51.8)
Yes	183 (48.2)
Marital Status	
Married	269 (70.8)
Single	111 (29.2)
Family Support	4.2 (0.8)
Low (1–2 point)	10 (2.6)
Medium (3 point)	64 (16.8)
High (4–5 point)	306 (80.5)
Work‐related characteristics	
Ward	
General ward	126 (33.2)
ICU	202 (53.2)
Emergency room	52 (13.7)
Length of service in nursing (years)	8.8 (8.5)
< 6 years	195 (51.3)
≥ 6 years	185 (49.7)
Shift Timing	
Only day shift	89 (23.4)
Only night shift	174 (45.8)
Day and night shift	117 (30.8)
Participation in Stress‐Related Education	
Yes	177 (46.6)
No	203 (53.4)

### 3.2 Levels of Perceived Stress, Self‐Efficacy and Burnout Among Nurses

Participants' mean scores for perceived stress and self‐efficacy were 24.9 (SD = 7.4) and 25.8 (SD = 5.5), respectively. The mean score for burnout was 25.9 (SD = 5.8). Most participants (43.7%) demonstrated moderate burnout. Details are presented in Table 2.

**TABLE 2:** Perceived stress, self‐efficacy and burnout among nurses (*n* = 380).

Variable	Mean (SD)/*N* (%)
Perceived stress	24.9 ± 7.4
Self‐efficacy	25.8 ± 5.5
Burnout	25.9 ± 5.8
Low	103 (27.1)
Moderate	166 (43.7)
High	111 (29.2)

Table 3 presents results of the bivariate correlations among perceived stress, self‐efficacy, and burnout. The results indicate that higher levels of perceived stress were significantly associated with lower levels of self‐efficacy (*r* = −0.655, *p* < 0.001) and higher levels of burnout (*r* = 0.664, *p* < 0.001). Moreover, higher levels of self‐efficacy were significantly associated with lower levels of burnout (*r* = −0.548, *p* < 0.001).

**TABLE 3:** Bivariate correlation among perceived stress, self‐efficacy and burnout among nurses (*n* = 380).

Variable	Perceived stress	Self‐efficacy	Burnout
Perceived stress	1	−0.655 (< 0.001)	0.664 (< 0.001)
Self‐efficacy	—	1	−0.548 (< 0.001)
Burnout	—	—	1

### 3.3 Mediation Analyses of the Influence of Self‐Efficacy on the Relationship Between Perceived Stress and Burnout

**FIGURE 1:**
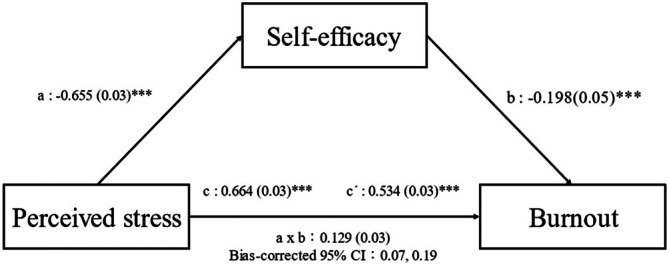
Mediation effects of self‐efficacy on the relationship between perceived stress and burnout. (a) effects of perceived stress on self‐efficacy. (b) effects of self‐efficacy on burnout after adjustment for perceived stress and covariates; (c) total effects of perceived stress on burnout. (c′) direct effects of perceived stress on burnout after adjusting for self‐efficacy and covariates. a × b: mediation effect of self‐efficacy on the relationship between perceived stress and burnout. The parameter estimates were presented as B (SE). ***p < 0.001. CI, confidence interval.

Figure 1 illustrates the results of our analysis of whether self‐efficacy acts as a mediator between perceived stress and burnout. As expected, the total effect of perceived stress on burnout was significant (*c* path: *B* = 0.664, *p* < 0.001). Furthermore, these effects were mediated by self‐efficacy (a × b: *B* = 0.129, 95% bias‐corrected bootstrap CI: 0.07 to 0.19), indicating that perceived stress indirectly influenced burnout by reducing self‐efficacy among nurses. Even after accounting for self‐efficacy and other covariates, the effects of perceived stress on burnout remained significant (*c’* path: *B* = 0.534, *p* < 0.001), suggesting a partial mediation. Additionally, the effects of perceived stress on self‐efficacy (*a* path: *B* = −0.655, *p* < 0.001) and of self‐efficacy on burnout (*b* path: B = −0.198, *p* < 0.001) were significant after controlling for perceived stress and other covariates.

### 3.4 Stratified Analysis of Relationships Between Perceived Stress, Self‐Efficacy and Burnout Among Nurses

Stratified analyses were used to examine the proportion of the indirect effect of self‐efficacy on the association between perceived stress and burnout within the subgroups defined by each variable. The findings demonstrated statistically significant proportions of the indirect effect of self‐efficacy, surpassing 30% in two subgroups: participants aged > 40 years (indirect effect = 0.280, *p* = 0.029) and those exclusively working day shifts (indirect effect = 0.259, *p* = 0.018). The details are provided in Table 4.

**TABLE 4:** Stratified analysis of path modelling for perceived stress, self‐efficacy and burnout among nurses (*N* = 380).

Variables	PS → Burnout		PS → Burnout		PS → SE → Burnout		Proportion to indirect effect
	Crude effect	*p* value	Direct effect	*p* value	Indirect effect	*p* value	
Demographics							
Age							
< 30 years	0.689	< 0.001	0.582	< 0.001	0.108	0.123	15.67
30–40 years	0.613	< 0.001	0.622	< 0.001	−0.009	0.927	1.47
> 40 years	0.634	< 0.001	0.354	0.002	0.280	0.029	44.16
Gender							
Female	0.668	< 0.001	0.549	< 0.001	0.119	0.026	17.81
Male	0.573	0.004	0.297	0.130	0.276	0.220	48.17
Educational level							
Junior college	0.658	< 0.001	0.503	0.001	0.155	0.327	23.56
Bachelor	0.676	< 0.001	0.582	< 0.001	0.094	0.122	13.91
Master	0.609	< 0.001	0.362	0.025	0.247	0.062	40.56
Religious Belief							
No	0.683	< 0.001	0.569	< 0.001	0.115	0.111	16.84
Yes	0.644	< 0.001	0.500	< 0.001	0.144	0.054	22.36
Marital Status							
Married	0.660	< 0.001	0.510	< 0.001	0.149	0.015	22.58
Single	0.652	< 0.001	0.597	< 0.001	0.055	0.567	8.44
Family Support							
Low (1–2 point)	0.751	0.012	0.093	0.668	0.658	0.108	87.62
Medium (3 point)	0.447	< 0.001	0.390	0.002	0.057	0.654	12.75
High (4–5 point)	0.662	< 0.001	0.571	< 0.001	0.091	0.114	13.75
Work‐related characteristics							
Ward							
General ward	0.677	< 0.001	0.492	< 0.001	0.185	0.041	27.33
ICU	0.681	< 0.001	0.565	< 0.001	0.116	0.102	17.03
Emergency room	0.474	< 0.001	0.415	0.014	0.059	0.678	12.45
Shift timing							
Only day shift	0.715	< 0.001	0.456	< 0.001	0.259	0.018	36.22
Only night shift	0.604	< 0.001	0.539	< 0.001	0.065	0.395	10.76
Day and night shift	0.654	< 0.001	0.535	< 0.001	0.119	0.205	18.20
Participation in stress‐related education							
Yes	0.659	< 0.001	0.488	< 0.001	0.170	0.025	25.80
No	0.668	< 0.001	0.571	< 0.001	0.097	0.171	14.52

Abbreviations: PS, perceived stress; SE, self‐efficacy.

## 4 Discussion

This study highlights the mediating role of self‐efficacy in the relationship between perceived stress and burnout among nurses in acute and critical care settings. Higher perceived stress was associated with lower self‐efficacy, which was in turn associated with higher burnout. Although statistically significant, these associations should be interpreted as correlational rather than causal, given the cross‐sectional design. The mediating effect of self‐efficacy was partial, as the direct path from perceived stress to burnout remained strong, indicating that self‐efficacy represents an important but not exclusive mechanism within this pathway. Burnout thus remains a multifactorial phenomenon influenced by both psychological and organisational factors.

These findings are consistent with prior studies conducted in China and Poland, which have demonstrated that elevated perceived stress is positively related to burnout among nurses [2, 33]. Nurses in acute and critical care contexts routinely face heavy workloads, complex decision‐making, emotional labour, and constrained resources, which elevate stress and increase burnout risk [18, 34]. Consistent with Bandura's Self‐Efficacy Theory (1986), nurses with higher self‐efficacy perceive challenges as manageable rather than threatening, thereby buffering the impact of stress. These results are consistent with prior studies conducted in diverse healthcare contexts in China, Spain, and Poland, which indicate that lower self‐efficacy and less perceived control exacerbate stress and burnout [22, 35, 36].

Although the mediating effect of self‐efficacy was significant, its magnitude was modest, suggesting that self‐efficacy alone does not fully account for how stress contributes to burnout. Other psychological and organisational factors, such as coping strategies, resilience, staffing adequacy and leadership support, likely interact within this pathway and should be included in future explanatory models. Nonetheless, even small psychological effects may have meaningful implications in high‐intensity care settings, where incremental resilience resources can reduce emotional exhaustion and turnover.

Subgroup analyses indicated that the mediating effect of self‐efficacy was stronger among nurses aged over 40 years and those working day shifts. These findings may reflect the cumulative professional experience, more stable work routines, and stronger peer networks of senior or day‐shift nurses, which enhance confidence and coping ability, and perceived control [37, 38, 39]. Self‐efficacy tends to increase with age and clinical experience, as repeated exposure to complex situations strengthens professional competence and adaptive coping skills [40, 41]. In older nurses, higher self‐efficacy has been linked to lower perceived stress and reduced burnout risk [42]. Similarly, day‐shift nurses often report higher self‐efficacy than night‐shift nurses, possibly due to more consistent work schedules, better sleep quality and greater social interaction [39, 43, 44]. Night‐shift nurses, in contrast, face greater physical fatigue, limited access to peer and managerial support and lower confidence in managing critical situations [37]. These factors collectively suggest that sustained experience and consistent professional support contribute to stronger self‐efficacy, which in turn mitigates perceived stress and burnout [22, 45]. Nevertheless, these subgroup results should be interpreted cautiously due to smaller sample sizes and wide confidence intervals. Future multicentre studies with larger, balanced samples are needed to confirm whether these differences represent genuine effects or sampling variation.

Overall, this study reinforces that self‐efficacy serves as a valuable psychological resource for mitigating perceived stress and burnout, but interventions should integrate both individual and organisational approaches. Contextually tailored strategies, such as integrating resilience training into simulation‐based emergency programmes, establishing structured mentorship between senior and junior nurses and facilitating peer‐support rounds may help strengthen self‐efficacy and adaptive coping. These efforts should be supported by system‐level measures, including adequate staffing, effective leadership and a positive safety culture, to ensure sustainable improvements in nurse well‐being and patient outcomes in acute and critical care environments.

## 5 Limitations

This study had several limitations. First, its cross‐sectional design limits causal inference; the associations among perceived stress, self‐efficacy, and burnout should be interpreted as correlational rather than causal. Longitudinal or interventional studies are needed to verify temporal and causal relationships. Second, quota sampling from a single medical centre and a culturally specific population of Taiwanese nurses may limit the generalisability of the findings. Cultural and organisational characteristics such as hierarchical structures and collectivist work norms may influence how self‐efficacy functions within the stress–burnout pathway. Multicentre and cross‐cultural studies are needed to validate these findings across diverse healthcare systems. Third, the use of self‐report questionnaires may introduce potential recall and social desirability bias, and the absence of objective indicators (e.g., physiological stress markers, absenteeism or turnover data) may limit data triangulation. Fourth, this study examined self‐efficacy solely as a mediator. Other potentially influential factors such as coping style, organisational climate and social support were not included and further explain or modify the stress–burnout relationships. Finally, although the indirect effects were statistically significant, their magnitude was modest. In addition, subgroup analyses involved relatively small sample sizes, increasing the risk of Type I error. Future studies should employ larger, more balanced samples and consider conservative statistical adjustments such as Bonferroni or false discovery rate corrections to enhance robustness.

## 6 Conclusions and Recommendations

This study provides evidence that self‐efficacy is a significant, though partial, mediator in the relationship between perceived stress and burnout among nurses working in acute and critical care settings. The persistence of a strong direct association between perceived stress and burnout underscores the multifactorial nature of burnout and highlights the influence of both individual psychological resources and organisational conditions.

From a practice perspective, interventions aimed at strengthening self‐efficacy may contribute to mitigating burnout but should be implemented alongside system‐level strategies. Contextually appropriate approaches such as integrating resilience‐building into simulation‐based training, establishing structured mentorship within clinical teams, and promoting supportive leadership may be particularly relevant in high‐acuity environments. Organisational efforts to ensure adequate staffing, effective communication and a supportive work culture remain essential.

By identifying subgroups that may be at heightened risk, including older nurses and those working day shifts, this study provides direction for targeted preventive strategies. Future research should adopt longitudinal or interventional designs and include broader organisational and cultural factors to clarify causal pathways and inform evidence‐based strategies that support nurse well‐being and workforce sustainability in critical care contexts.

## Funding

This work was supported by the Ministry of National Defense‐Medical Affairs Bureau (MND‐MAB‐110‐097, MND‐MAB‐D‐115161, MND‐MAB‐109‐023), National Defense Medical University (MND‐MAB‐D‐115161).

## Disclosure

The authors disclose the use of GPT‐5 (accessed August 2025) for limited language‐related assistance, including grammar correction, sentence refinement and translation of selected phrases. All AI‐assisted content was critically reviewed, edited, and validated by the authors, who take full responsibility for the accuracy, integrity and originality of the manuscript.

## Ethics Statement

This study was approved by the Institutional Review Board of Tri‐Service General Hospital (B202005010) on 29 April 2020.

## Consent

Written informed consent was obtained from all participants prior to data collection, in accordance with the ethical standards approved by the IRB.

## Conflicts of Interest

The authors declare no conflicts of interest.

## Data Availability

The datasets generated and/or analysed during the current study are not publicly available due to participant privacy but are available from the corresponding author on reasonable request.
